# Trait-based community assembly and functional strategies across three subtropical karst forests, Southwestern China

**DOI:** 10.3389/fpls.2024.1451981

**Published:** 2024-09-09

**Authors:** Yong Jiang, Zhenqing Chen, Haili Lin, Rongxin Deng, Zhihui Liang, Yuling Li, Shichu Liang

**Affiliations:** Key Laboratory of Ecology of Rare and Endangered Species and Environmental Protection, Ministry of Education, College of Life Sciences, Guangxi Normal University, Guilin, China

**Keywords:** karst hills, forest types, traits strategy, environmental gradient, community assembly

## Abstract

**Background:**

Variations in community-level plant functional traits are widely used to elucidate vegetation adaptation strategies across different environmental gradients. Moreover, studying functional variation among different forest types aids in understanding the mechanisms by which environmental factors and functional strategies shift community structure.

**Methods:**

Based on five plant functional traits, including four leaf and one wood trait, for 150 woody species, we analyzed shifts in the community-weighted mean trait values across three forest types in a karst forest landscape: deciduous, mixed, and evergreen forests. We also assessed the relative contributions of stochastic processes, environmental filtering, and niche differentiation to drive community structure using a trait-based null model approach.

**Results:**

We found marked changes in functional strategy, from resource acquisition on dry, fertile soil plots in deciduous forests to resource conservation on moist, infertile soil conditions in evergreen forests. The trait-based null model showed strong evidence of environmental filtering and convergent patterns in traits across three forest types, as well as low niche differentiation in most functional traits. Some evidence of overdispersion of LDMC and LT occurred to partially support the recent theory of Scheffer and Van Nes that competition could result in a clumped pattern of species along a niche axis.

**Discussion:**

Our findings suggest a change in environmental gradient from deciduous to evergreen forest, together with a shift from acquisitive to conservative traits. Environmental filtering, stochastic processes, niche differentiation, and overdispersion mechanisms together drive community assembly in karst forest landscapes. These findings will contribute to a deeper understanding of the changes in functional traits among karst plants and their adaptive strategies, with important implications for understanding other community assemblies in subtropical forest systems.

## 1 Introduction

Functional traits are composed of a series of physiological, ecological, and behavioral characteristics that strongly influence individual recruitment, reproduction, and mortality. These trait attributes gradually developed through the long evolutionary history of nonindigenous plant species, which have adopted convergent and divergent ecological strategies for resource capture and allocation (Nock et al., 2016; Long et al., 2023). Some traits partially reflect an individual species’ adaptation to changing environmental gradients (McIntyre et al., 1999), which makes them important for exploring species maintenance and assembly structure (Violle et al., 2012). For example, in resource-rich, dry habitats, short-lived species are grouped as fast resource acquisition species that invest in cheap, transitory leaves, high specific leaf area, and low wood density that afford a fast return on investment (Zhang et al., 2021; Wang et al., 2023). In contrast, shade-tolerant species in harsh, wet habitats display conservative strategies that showcase slow growth rates and ultimately invest in long-lasting tissues and high leaf dry matter content that aid in their defense against herbivores, pathogens, and physical damage (Weiher et al., 2011; Zhang et al., 2021; Wang et al., 2023). Together, these trade-offs in functional traits along resource-use environmental gradients are not well understood and could potentially explain key differences in community assembly. Karst regions are unique and complex ecosystems that have not been extensively studied and are characterized by exceptional geological, environmental, and floristic conditions. The landscape features steep, irregular soil surfaces with rocky outcrops, where soil properties are influenced by the water dissolution of soluble bedrock (Chen et al., 2013). Long-term dissolution and erosion have led to bare rock distributions and diverse topographies with fragmented structural attributes, limiting vegetation growth. Species in this region are often patchily distributed due to drought risks and nutrient-poor soils (Veress, 2020; Liu et al., 2021). The functional traits of these species are adapted to specific strategies that counteract drought stress (Wei et al., 2020). For instance, evergreen trees in karst areas typically exhibit lower specific leaf area and leaf area, along with higher leaf dry matter content and thickness, enabling them to develop drought-resistant characteristics suited to the stress of poor karst soils and limited water supply (Muscarella et al., 2020). Therefore, understanding community assembly mechanisms along environmental gradients from a functional trait perspective is crucial for elucidating the adaptive strategies of plant species in karst regions.

The environment acts as a filter for selecting viable strategies with optimal trait distribution that leads to trait convergence by controlling the spatial distribution of light, soil water, and nutrient gradients through geographic processes (Zhu et al., 2016). Furthermore, convergent local trait distributions tend to have similar resource requirements for survival that make competition more intense (Marteinsdóttir et al., 2018; Qiao et al., 2023). In this regard, intense competition is expected to exclude species with highly similar traits and form divergent distributions (Bernard-Verdier et al., 2012; Xu et al., 2018; Zhang et al., 2020). However, competitive exclusion could also contribute to functional clustering (i.e., overdispersion pattern) by excluding functionally similar species with low competitive abilities (Yan et al., 2012; Li et al., 2015). Ultimately, these changes demonstrate that trait distributions within communities allow for the quantification of distinct screening processes and restrictions on assembly structure along environmental gradients (Freschet et al., 2011; Violle et al., 2012; Chen et al., 2022; Fu et al., 2023). Together, they tend to reflect the important fundamental niche axes that allow species to differentiate and assess trait convergence, divergence, and overdispersion patterns (Violle and Jiang, 2009; Marcilio-Silva et al., 2016).

Previously, some studies have indicated that trait-mediated abiotic filtering plays a pivotal role in subtropical forest ecosystems (Liu et al., 2021; Wang et al., 2023; Vleminckx et al., 2023), while other research suggests that niche differentiation is the main ecological driver in some tropical forest ecosystems (Meilhac et al., 2019; Wang et al., 2022a; Nomura et al., 2023; Wang et al., 2023; Han et al., 2023). Likewise, stochastic processes, to some extent, have also been proposed as potential interpretations for the assembly and ecological community structure (Czortek et al., 2021; Chen et al., 2023; Lepš and De Bello, 2023). In particular, Uriarte et al. (2010) found that plant traits tend to exhibit an overdispersion pattern as niche differentiation among taxa increases under stressed conditions. However, it remains unclear how specific forest regions, such as the Karst Forest, respond to functional traits along different resource-use environmental gradients.

In this work, we used three natural forest types with species-rich karst-hill plant communities to investigate the determinants of plant community assembly. The communities covered a range from deciduous broad-leaved forests (deciduous forests), mixed evergreen and deciduous broad-leaved forests (mixed forests), and evergreen broad-leaved forests (evergreen forests) with their own unique ecological and functional attributes. Deciduous dominance forests have been found to contribute significantly to seasonal changes that bring about striking transformations in the landscape by adopting a falling strategy to face the harsh winters (Letcher et al., 2015). With the mixed forests, it acts as a transition between the deciduous and evergreen species and hosts a fascinating blend of flora. Additionally, it often exhibits contrasting ecological strategies to cope with the turnover of environmental factors (Ge et al., 2019). In comparison, evergreen forests are characterized by the dominance of evergreen trees that present an acquisitive strategy with strong texture leaves, as well as low photosynthesis rates with slow growth rates to resist stressed habitats (Lv et al., 2022). Taking into account these different forest types, it is necessary to explore the ecological strategies of these forests to elucidate their resource utilization capacity in karst forest landscapes. Thus, we explored differences in five CWM_traits_ with environmental gradients across three forest types to answer two questions below: (1) How do the functional traits of plant communities and their adaptation strategies vary by forest types, and (2) Are assembly-based traits structured by different processes with these specific forest types? We hypothesized that functional community-level responses and strategy variations to resource availability changes would strongly depend on community relationships with the affected resource. We also expected that there would be different factors, including abiotic and biotic, that modify assembly processes with different forest types. Ultimately, this process will permit us to discover which distribution trait patterns allow for the assembly of communities with changing habitat gradients.

## 2 Materials and methods

### 2.1 Study location

The study area was located in the Guilin karst hills region in the northeastern Guangxi Zhuang Autonomous Region, China. Elevation ranges from 180 to 230 m above sea level. The region has a typical subtropical monsoon climate with hot and wet summers and cold and dry winters. Mean annual precipitation ranges from 1,814 to 1,941 mm, and rainfall is unevenly distributed throughout the year, with 62% of the annual rainfall from April to July. The mean annual air temperature is 17.8°C–19.1°C, with a free frost period of 309 days (data from the China Meteorological Data Service Center, http://data.cma.cn). The soil layer is thin and discontinuous, primarily found in rock fractures (Liang et al., 2020). The vegetation in the study area had naturally regenerated after significant industrial activities in 1958–1960 (Huang, 2012), and the government banned the felling of trees and promoted the natural recovery of vegetation.

In this paper, we identified three typical vegetation types: deciduous forests, mixed forests, and evergreen forests as the sample points according to source publications or local flora reference guides, for instance, the Vegetation of China, the Forest of Guangxi (Guo et al., 2020; Wen et al., 2022), and research on karst forests (Su and Li, 2003). In addition, we applied the following statistical tests to the cross-validation data: (1) Importance value (IV) was used as a comprehensive quantitative index to rank the dominant plant species per forest type (Jiang et al., 2021). (2) Correspondence analysis (CA) method was performed on species importance value data to distinguish three different forest types. (3) Means of analysis of similarities (ANOSIM; Clarke, 1993) was applied using the Bray–Curtis distance matrix to test for statistically significant difference between two forest types. A more detailed supplementary information is shown in **Supplementary Table 1** and **Supplementary Figures 1**–**3**. The local terrain, environmental factors, and dominant species are different for each of these forest types. Deciduous forests are mainly made up of lianas, shrubs, and a range of shade-intolerant deciduous species. This area is characterized by low water levels, high soil nutrient content, and a relatively thick soil layer (< 35 cm). Shade-intolerant deciduous dominant species were *Celtis sinensis*, *Choerospondias axillaris*, and *Boniodendron minus*. The environment of the mixed forests changed with gradually increasing soil water content and intermediate soil nutrient levels. These forests were characterized by highest rock exposure and a relatively shallow soil layer (< 30 cm). The dominant species comprise *Quercus glauca*, *Zelkova schneideriana*, *Boniodendron minus*, *Alchornea trewioides*, *Callicarpa bodinieri*, and *Mallotus philippensis*. Evergreen forests are distributed in areas with poorer soil conditions, characterized by low soil nutrient content, rock exposure, and highest soil water content. These forests tend to be located on relatively flat slopes with the thickest soil layer (< 37 cm) compared with the other two forest habitats. The main tree species include *Quercus glauca*, *Mallotus philippensis*, *Pittosporum planilobum*, and *Alchornea trewioides*. Basic information on species features and soil terrain for different forest types is presented in **Table 1**.

**Table 1:** Basic information on different forest types.

Parameters	Deciduous forests		Mixed forests		Evergreen forests	
	Tree layer	Shrub layer	Tree layer	Shrub layer	Tree layer	Shrub layer
Deciduous/evergreen species richness	22/16	29/32	30/32	29/34	29/35	38/34
Deciduous/evergreen species abundance	276/245	864/1,555	235/405	529/436	598/1,635	2192/2,576
Deciduous/evergreen important value (%)	64.76/35.24	65.62/34.38	45.50/54.50	49.90/50.10	26.29/73.71	48.71/51.29
Convexity	− 8.5 to 12.3		− 13.3 to 8.6		− 10.2 to 10.3	
Elevation (m)	184–228		179–244		195–240	
Slope	15.06°–59.38°		24.71°–47.63°		17.96°–45.88°	

### 2.2 Field sampling

We did field surveys during three separate periods: July to September in 2019, 2021, and 2022. For each forest type, 25 parallel plots (20 m × 20 m) with a buffer distance of > 10 m between adjacent plots were randomly selected in some sections under the same slope (i.e., Mao Village in Lingchuan County, Long Village in Yongfu County, and Beitou Village in Yangshuo County). Each 20 m × 20 m plot was further divided into four 10 m × 10 m subplots for surveying. The deciduous forests have two standing strata: the first is mainly composed of trees that are 4–8 m tall, and the other is mainly dominated by shrubs and vines. The mixed forests have two standing layers. The first is made up of sparse woody species with a plant height ranging from 8 to 12 m. The second consists of a sparse understory with shrubs and herbaceous perennials. The evergreen forests contain three vertical layers; large trees above 12 m form the first layer with a relatively closed canopy. The second encompasses juvenile trees of diverse sizes, and the third contains typical understory shrub trees and herbaceous perennials. Within each 10 m × 10 m subplot, we enumerated, measured, and taxonomically identified all free-standing woody trees with a diameter of ≥ 1 cm at 1.3 m height. Species nomenclature followed Flora of China (English version, https://www.iplant.cn/focnt.cn) and expert identification. In total, we sampled 11,546 woody stems that belong to 150 species, 104 genera, and 47 families.

### 2.3 Measurement of environmental factors

Environmental variables, including physical and chemical soil properties, were measured at each 20 m × 20 m sampling plot per forest type. At each 20 m × 20 m sampling plot, five soil samples were collected randomly from the 20 cm of topsoil at the four corners and center of the sampling plot. Leaf litter, rocks, and other major debris were removed before sampling. Approximately 10 g of each fresh soil sample was dried at 105°C for 6 h to measure soil water content (SWC, %). Soil thickness (ST, cm) was measured using a 1.5-m steel bit hammered into the parent rock at each soil location. The other soil samples were dried naturally at room temperature within a month and then sieved through a 2-mm mesh sieve for further laboratory experimentation. Soil properties including soil pH, total nitrogen (TN, g/kg), available nitrogen (AN, mg/kg), available phosphorus (AP, mg/kg), and water-soluble calcium (Ca, g/kg) were quantified according to standard soil agricultural and chemical analysis protocols (Bao, 2000). Understory irradiance was estimated using hemispherical canopy photographs taken at each 10 m × 10 m sampling subplot, 1.5 m above ground level, with a fisheye lens mounted on a camera (Nikon D80). A canopy cover was obtained from each photo occupying the proportion of closed-canopy pixels by a software Gap Light Analyzer (Frazer et al., 2000). Canopy openness (CO, %) was calculated as one-canopy cover. CO in deciduous forests was estimated during the period of leaf fall, and the other two forest types were obtained as usual. The rock–bareness ratio (BRR, %) was visually estimated as the proportion of the aboveground rocks to the ground surface within each 10 m × 10 m sampling subplot. Elevation and other topographic data were automatically acquired using the JRBP Geographic Information System (GIS).

### 2.4 Measurement of functional trait

We sampled each individual to quantify five functional traits for 150 species identified at different forest types, referring to the criteria of Pérez-Harguindeguy et al. (2013). These five functional traits were leaf area (LA, cm^2^), specific leaf area (SLA, cm^2^/g), leaf dry matter content (LDMC, g/g), leaf thickness (LT, mm), and wood density (WD, g/cm^2^), representing a wide range of plant ecological utilization strategies related to competitiveness, chemical defenses, drought stress, and light capture (Pérez-Harguindeguy et al., 2013). For example, SLA and LA with LDMC are a series of traits partly belonging to the leaf economic spectrum, which is affected by light intensity, resource acquisition, and water balance (Ma et al., 2021; Wang et al., 2022b). LT is associated with defensive ability, tolerance to photosynthetic intensity, and water and material storage (Schmitt et al., 2022). WD has been thought to be orthogonal to other leaf traits as an important determinant of xylem water transport, drought tolerance strategies, and mechanical support (Chave et al., 2009; Wu et al., 2021). For each individual, three recently mature leaves and three 1–2-cm diameter branches were collected from the upper branches. Leaves were not necessarily “sun-exposed”, as many species grow to their maximum size in the understory and never experience full sunlight exposure. If the canopy height reached 10 m or more, leaf samples were picked from sun-exposed branches using high-branch scissors. For understory species, leaves were selectively sampled from the upper parts of the plants (Pérez-Harguindeguy et al., 2013). Collected leaves and branches were first placed into sealed plastic bags, then transferred to a big woven polypropylene bag, and finally shipped to the laboratory for further measurement within 12 h of collection. In total, 34,827 leaves were sampled, belonging to 11,546 individuals. LA was detected by a YMJ-C scanner (Tuopu, Zhejiang, China) with a corresponding self-developed software system. SLA was calculated from LA and LDMC measurements (at 70°C for 48 h). The LDMC was determined by calculating the ratio of the mass after oven-drying to the initial fresh mass. LT was detected with calipers using three replicates for each sample (precision: 0.05 mm). To characterize species wood density, we collected three branches per tree. The pith, phloem, and bark were removed using a lopper. Fresh volume was measured by water displacement, followed by determination of dry mass after oven-drying at 70°C for 6 h until a constant weight was reached. WD was calculated based on the ratio of oven dry mass to fresh volume (Bu et al., 2014).

### 2.5 Statistical analyses

To better compare and analyze trait variation at different forest types, we calculated the community weighted mean (CWM) values for 25 plots in each one on the basis of a species-trait dataset and a plot-species abundance dataset. The CWM values were weighted by plot abundance and summed across all species traits within the plot, representing a community-level mean trait value and emphasizing the importance of the more abundant species. The formula is as follows:


$$CWM=\sum_{i=1}^{S}W_{i}\times X_{i}$$

Where *S* represents species number at a plot within each forest type, W*_i_* is the relative abundance of species *I*, and *X_i_* is the functional trait value of species *i* at each plot within each one (Jiang et al., 2015). We then applied a one-way analysis of variance and Tukey’s multiple comparison treatments to evaluate differences in both community-level traits and environmental factors between two groups of different forest types (Kazakou et al., 2022).

We have used a trait-based null model approach to detect nonrandom assembly processes by calculating two metrics within plots relative to simulated communities at different forest types. The first metric was the range of traits (metric 1), which was used to detect environmental filtering (Jung et al., 2010; Lhotsky et al., 2016). The second metric was the variation coefficient of the nearest-neighbor distance (CV_NND, metric 2) between traits, which was used to test for niche differentiation (Jung et al., 2010; Kohli et al., 2018). Null models were constructed using a two-step procedure to detect trait distributions. First, we randomly selected species without replacement from a total species pool, including recorded species at each forest type. Second, we allocated each previously selected species a random species mean trait. Next, 999 random communities were constructed for species richness within plots of different forest types. For metric 1 (range), an observed trait value less than the null expectation indicates that environmental filtering had a significant difference in trait distribution. For metric 2 (CV_NND distance), an observed trait value lower than expected under the null model (i.e., trait values more evenly spaced) indicates niche differentiation (Kraft et al., 2008). In order to estimate observed trait distribution deviation direction from the those expected by chance, the standardized effect size ( $SES_{I}$ ) was examined for each $plot_{I}$ within each one, which was conducted using the “picante” package in R (Kembel et al., 2010):


$$SES_{I}=\frac{I_{obs}-I_{null}}{\sigma_{null}}$$

Where $I_{obs}$ is the observed trait mean value in $plot_{I}$ , $I_{null}$ is the null model trait mean value in $plot_{I}$ , and $\sigma_{null}$ is the null model standard deviation in the $plot_{I}$ . The positive effect showed an observed value greater than the average expected value, and the negative effect indicated an observed value lower than the average expected value. Here, to avoid potential misleading results from SES calculations when trait distribution does not conform to the normal distribution, we applied a log transformation to the data before calculating the SES values (Botta-Dukát, 2018). Finally, we used a nonparametric Wilcoxon statistical test to assess the significant difference between the observed trait mean distribution and the null trait mean distribution among plots at different forest types, where the alpha was set to a 95% confidence interval with a *p* < 0.05 set as significant. The nonparametric Wilcoxon statistical analysis was tested using the “stats” package in R (version 4.3.2, R Core Team 2024).

## 3 Results

### 3.1 Change in CWM_traits_ and environmental variables at different forest types

**Figure 1:**
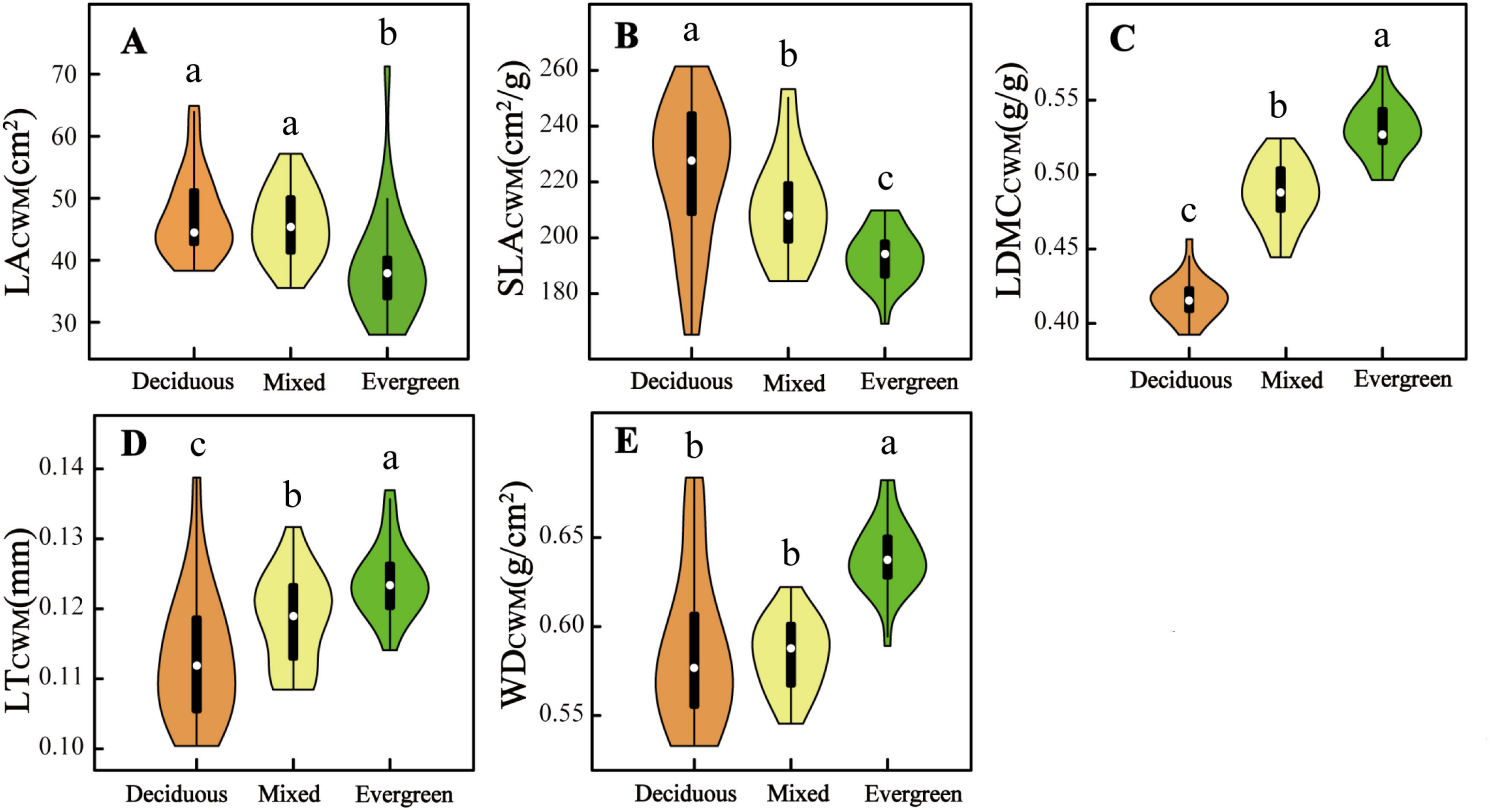
Difference in five community-weighted **(A–E)** mean functional traits across three forest types. The distinct letters (a–c) stand for significant differences among the three forest types (p < 0.05). LA_CWM_, community-weighted mean leaf area; SLA_CWM_, community-weighted mean specific leaf area; LDMC_CWM_, community-weighted mean leaf dry matter content; LT_CWM_, community-weighted mean leaf thickness; WD_CWM_, community-weighted mean wood density.

**Figure 2:**
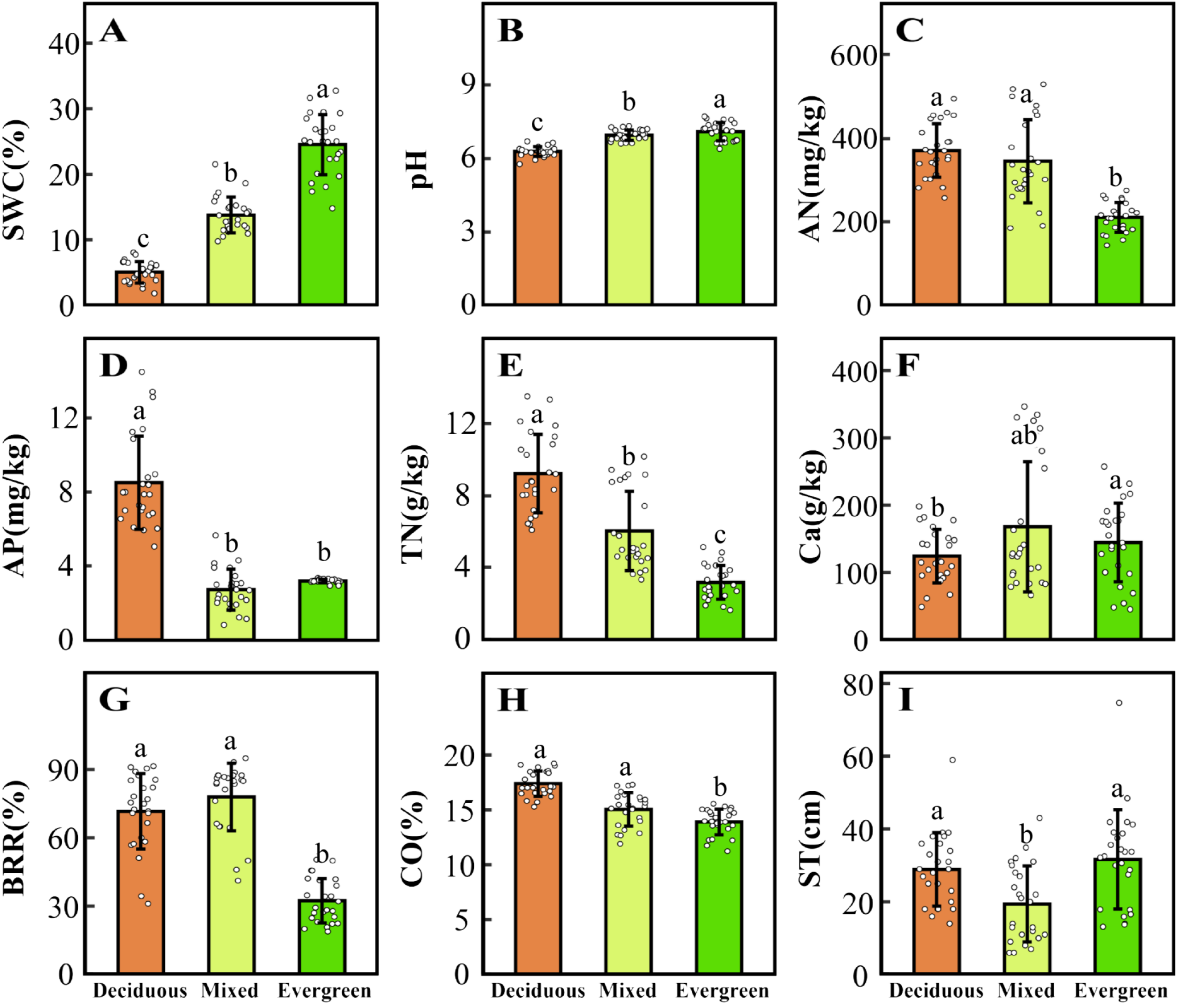
Changes in environmental factors **(A–I)** across three forest types. The distinct letters (a–c) stand for significant differences among the three forest types (p < 0.05). SWC, soil water content; pH, pondus hydrogenii; AN, available nitrogen; AP, available phosphorus; TN, total nitrogen; Ca, water-soluble calcium; BRR, rock-bareness ratio; CO, canopy openness; ST, soil thickness.

Five CWM_traits_ and nine environmental factors significantly differ in deciduous, mixed, and evergreen forests (*p <* 0.05, **Figures 1**, **2**). Among them, the SLA_CWM_, LDMC_CWM_, and LT_CWM_ showed significant differences across three forest types (*p* < 0.05, **Figures 1B–D**, respectively). In contrast, no significant differences were found in LA_CWM_ and WD_CWM_ between deciduous and mixed forests (*p* > 0.05), but these two differed from evergreen forests (*p* < 0.05, **Figures 1A, E**). The soil factors SWC, pH, and TN revealed significant differences among the three forests (*p* < 0.05, **Figures 2A, B, E**). The AN and CO were significantly lower in evergreen forests than in deciduous and mixed forests (**Figures 2C, H**). Additionally, the Ca content for deciduous and evergreen forests was lower than that for mixed forests, and significant differences were exhibited between deciduous and evergreen forests (*p<* 0.05, **Figure 2F**). Furthermore, the AP for deciduous forests was significantly greater than for mixed and evergreen forests (*p <* 0.05, **Figure 2D**), whereas BRR for evergreen forests was obviously lower than for mixed and deciduous forests (*p <* 0.05, **Figure 2G**). In addition to these attributes, the ST was markedly lower in mixed forests compared to deciduous and evergreen forests, but no significant differences were found between these two (*p* > 0.05, **Figure 2I**).

### 3.2 Null model test at different forest types

**Figure 3:**
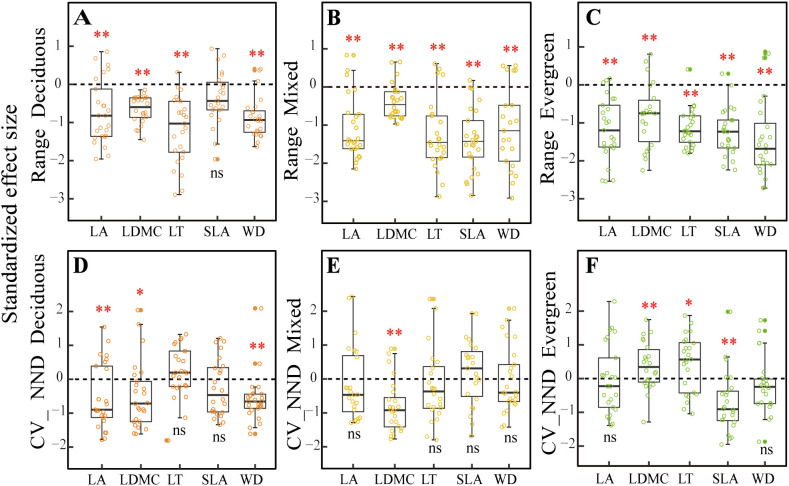
Comparison of null models**(A–F)** for five functional traits across three forest types. Note: The asterisks represent that the observed community significantly differs from the random community under the Wilcoxon signed-rank test (^*^ *p* < 0.05; ^**^ *p* < 0.01; ns, difference is not significant). LA, leaf area; LDMC, leaf dry matter content; LT, leaf thickness; SLA, specific leaf area; WD, wood density; CV_NND, coefficient of variation of the nearest-neighbor distance.

For the trait range (metric 1) that was used to detect environmental filter (**Figures 3A–C**), we found that the SES values for all the traits were significantly different from zero at different forest types (*p<* 0.01), except that SLA showed no significant difference from zero in deciduous forests (*p* > 0.05, **Figure 3A**) but was significantly different from zero in mixed and evergreen forests (*p<* 0.01, **Figures 3B, C**).

For the CV_NND (metric 2) that was used to test for niche differentiation (**Figures 3D–F**), the SES value of LA, LDMC, and WD were all significant differences from zero in deciduous forests (*p <* 0.05, **Figure 3D**), whereas only the SES values of LDMC were significant differences from zero in mixed forests (*p <* 0.05, **Figure 3E**). Still, SLA did not present a significant difference from zero between deciduous and mixed forests (*p* > 0.05, **Figures 3D, E**) but was significantly different from zero in evergreen forests (*p <* 0.01, **Figure 3F**). The LDMC and LT standard deviations for the nearest-neighbor distances were markedly greater than zero in evergreen forests (*p<* 0.05, **Figure 3F**).

## 4 Discussion

### 4.1 CWM_traits_ response to environmental conditions at different forest types

With the changes in soil water and soil nutrient content, topographical factors (such as rock-bareness rate, slope, and elevation), and light conditions across three forest types, the CWM_traits_ shifted accordingly (**Figures 1**, **2**; **Supplementary Figure 4**). The SLA_CWM_ was the highest in deciduous forests, followed by mixed and evergreen forests, whereas the LDMC_CWM_ displayed the opposite trend. It is well known that SLA and LDMC are considered useful proxies for axes of resource capture and use (Wang et al., 2023). This result fits our expectations, as deciduous habitat had high soil AN, AP, and TN (**Figures 2C–E**). Deciduous trees tend to have relatively higher SLA but lower LDMC than evergreen trees, reflecting the “fast” vs. “slow” life-history strategies of these two tree groups (Wang et al., 2023). Similarly, we found relatively higher LA_CWM_ in both the deciduous and mixed forests compared with evergreen forests, in line with the greater CO in forests dominated by deciduous taxa (Schönbeck et al., 2015; Lusk et al., 2019). This indicated deciduous species had a strong ability to capture light resources and a higher relative growth rate by increasing LA (Cheng et al., 2022).

Other characteristics are also changed by variable forest habitats. For example, LT_CWM_ was higher in more evergreen forest plots and also positively related to SWC (**Figures 1D**, **2A**; **Supplementary Figures 4**, **5**). Thicker leaves are often related to water and material storage (Schmitt et al., 2022), adopting a survival strategy for plants in arid and poor environments. Similar to Kröber’s findings, evergreen species tend to have thicker leaves, which help the tissue maintain turgor pressure and avoid water stress (Kröber et al., 2015). In contrast, deciduous species may use better drought-coping strategies to enhance water use efficiency; for instance, Vargas et al. (2021) found that deciduous species have higher SLA in drought environments, thus providing enhanced root foraging capacity for water under drought conditions (Wellstein et al., 2017). Additionally, WD_CWM_ had the lowest values in deciduous forests because of low water and high soil nutrients, which indicates that species with low WD tend to grow rapidly because of low investment in structural material. Conversely, species with the highest WD were typically found in evergreen forests where soil nutrients, such as AP, AN, and TN, were significantly lower than the other two forest habitats, which suggests that these species have enhanced resistance to structural damage and mortality caused by drought. In summary, we found that functional traits shift with changing environmental gradients at different forest types in the karst forest landscape and reflect a general transition from a fast-growing strategy toward a more resource-conservative strategy.

### 4.2 Environmental filtering of different traits at different forest types

Our results indicate that environmental filtering deterministic processes are a major contributor to community assemblies in karst forests, and a significantly reduced range of five functional compared to null expectations was observed (**Figures 3A–C**). At the same time, we found that SWC, AP, AN, CO, and slope significantly influenced five CWM_traits_ in deciduous forests, while SWC, TN, AP, BRR, and slope had a strong impact on them in evergreen forests, as evidenced by the correlation of environmental factors with traits in the CCA ordination analysis (**Supplementary Figure 5**), which further well supported the driving role of environmental filters in the assembly of karst forest communities. These findings were in line with the previous studies conducted in different forest types. For example, higher LA and lower LDMC, as well as WD in deciduous dominance forests, provide strong evidence that light availability and soil phosphorus are important environmental filters (Huang et al., 2018; Shivaprakash et al., 2018). Huang et al. (2021) also found patterns of shifts in functional trait distribution such as SLA, LDMC, and WD were strongly influenced by the combined impacts of environmental and terrain factors within the subtropical evergreen broadleaved forests. Similarly, in karst forest systems, some experimental studies have emphasized that plants showed convergent distribution of functional traits (SLA) as stressed environmental conditions (such as the limited water and lower soil phosphorus) become more evident (Zhang et al., 2020; Liu et al., 2021; Wang et al., 2022b). In the present study, our findings clearly support the hypothesis that environment filtering may exist in three distinct forest types due to resource availability. This may be limited in karst, diverse topographies with fragmented structural attributes because typical rock-soil structures cause serious differences in soil water-holding capacity and its nutrient content under different forest habitats. Specifically, at deciduous forest plots, SWC was the lowest and possessed a high bare soil surface that made intense competition for resources very unlikely. Thus, this trait convergence is likely the result of special karst habitat filtering (Chen et al., 2013). The mixed forests, a transition between deciduous and evergreen species, should exhibit contrasting ecological assembly mechanisms to be in a state of simultaneous coexistence under this complex habitat, such as competition intensity, nutrient availability, and the level of importance of habitat filtering (Purschke et al., 2013). However, in these mixed forests, environmental factors are still the prominent drivers in structuring community assembly. Ultimately, this is possible because fitness disparities between these strategies in this “comprehensive milieu” are likely to be less than special karst habitat differences. Here, we found in evergreen plots that SWC continued to increase (**Figure 2A**), while the bare ground cover decreased (**Figure 2G**), and the soil layer is the thickest with low light availability (**Figures 2H, I**), which would likely increase competition intensity. As a corollary, we should observe functional divergence during evergreen forests. For instance, Backhaus et al. (2021) showed that trait divergence becomes more common at later stages in evergreen habitats, mainly due to competition limiting the trait similarity of species. Nevertheless, we mainly observed that environmental factors are still part of the major mechanism in evergreen forests (**Figure 3C**). This result may be closely related to the low light availability, poor soil nutrients, and special local topography under this forest habitat. Together, these observations also support the theory that light, soil properties, and topographical factors operate as comprehensive environmental filters for plants with functional traits to tolerate special karst environmental conditions.

### 4.3 Niche differentiation of diverse traits at different forest types

For the CV_NND that was used to test niche differentiation, we observed that most functional traits displayed a stochastic pattern (**Figures 3D–F**) with weak-to-no evidence of niche differentiation across three different types. This may be attributed to the characteristics of the karst environment, which is prone to water shortages, has poor soil nutrient availability, and features unique local topography. Consequently, plant communities in karst areas tend to exhibit strong environmental filtering due to these harsh abiotic conditions (López-Martínez et al., 2013). We also discovered that stochastic processes, rather than niche differentiation, predominated in the infertile, dry soil environment for most of the studied traits. However, both environmental filtering and competitive exclusion may produce opposing influences on community composition, potentially canceling each other out and resulting in an overall stochastic outcome (Bernard-Verdier et al., 2012; Marteinsdóttir et al., 2018). For example, it is possible that both competitive ability differences for light in height and differences in preference for soil can cancel each other out, leading to a random pattern (Mayfield and Levine, 2010). Additionally, we identified some evidence of trait divergence in particular traits and plots. Specifically, divergence in LA, LDMC, and WD was noted in deciduous and mixed forest plots, likely driven by intense competition resulting from limited resources (such as water, soil nutrients, and local topography) (Buzzard et al., 2016; Blanchard et al., 2021; Li et al., 2021; Wu, 2023). Furthermore, the trait divergence for SLA in evergreen forests may also stem from distinct strategies for resource usage and capture (Mudrák et al., 2016; Doležal et al., 2019). We observed a significant change in environmental factors, moving from low water with rich soil nutrient content in deciduous forests to high water with poor soil nutrient content in evergreen forests. This change indicates that soil nutrient availability in evergreen-dominance forests is a limiting resource for SLA, resulting in intense competition as species strive to exploit a larger SLA functional trait space to enhance niche differentiation among co-occurring species.

Altogether, our findings concerning the overdispersion of traits in evergreen forests support the theory of Scheffer and Van Nes (2006) and are in contrast to classical niche theory (Tilman, 2004), where species exhibited weak competition once they have set up an even trait distribution. According to Scheffer and Van Nes’s theory, competition occurs within clusters of similar species. However, if there is symmetrical distribution and it does not result in the long-term exclusion of a species, a different outcome occurs. This process mostly occurs in stressful conditions where competitors have similar traits to one another and results in more intense competition along different niche axes (Yan et al., 2012). We observed the overdispersion of LDMC and LT in evergreen forests, which supports this hypothesis. This may be closely related to the stressful habitat of evergreen forests, characterized by low soil nutrient content, limited light availability. During forest sampling investigation, we found that evergreen species in both the tree and shrub layers occupied a large proportion of individuals compared to the other two forest types. The community structure was relatively complex, consisting of three vertical strata of trees, with the tree layer forming a closed canopy that established its own local habitat. We noted that species with a combination of higher LDMC and LT traits were more abundant in some plots than in others. Generally, species with more similar traits will be more competitive under limited resource conditions. These species with large abundance could allow them to flow over into the niches of other species through a kind of mass effect (Guélat et al., 2008; Yan et al., 2012) and hence rule out other species with neighboring niches and diverse trait combinations. For instance, in evergreen-dominant forests, deciduous species characterized by higher SLA, LA, and lower WD, LDMC, and LT were excluded by evergreen species possessing higher LDMC and LT, alongside lower SLA, LA, and WD. This combination of functionally equivalent traits (i.e., clumps of functionally equivalent traits) may hinder competitive exclusion and lead to an overdivergent trait distribution (Hubbell, 2001; Vialatte et al., 2010; Yguel et al., 2011; Yan et al., 2012).

### 4.4 Some ideas for restoring karst forests in SW China from a trait-based point of view

The functional trait approach has significantly advanced the field of community ecology, transforming it from a complex to a simple research process. Additionally, the development of functional ecology has enabled ecologists to accurately identify functional trait axes related to resource acquisition, forest regeneration, and environmental tolerance, linking functional diversity to ecological strategies, processes, and functions, thus providing a new tool for research that classical community ecology could not offer. Since different species possess different functional traits, they play diverse functional roles in different forest ecosystems. This work’s main focus should be on how to identify species with the optimal combination of functional traits for restoration and reconstruction based on the special karst habitat. This includes the selection of species with drought resistance, low nutrient tolerance, and adaptability to harsh conditions. By doing so, we can effectively assemble a species composition that is not only resilient to environmental stressors but also capable of supporting the overall health and functionality of the karst forest ecosystem.

## 5 Conclusions

This study revealed the adaptation of community-level functional traits shifts with changing environmental gradients at different forest types and reflects a general transition from a fast-growing strategy toward a more resource-conservative strategy. Environmental filtering, stochastic processes, niche differentiation, and overdispersion mechanisms together drive community assembly in karst forest landscapes. These findings will contribute to a deeper understanding of the changes in functional traits in karst plants and their adaptative strategies and have important implications for understanding other community assemblies in subtropical forest systems.

## Data Availability

The raw data supporting the conclusions of this article will be made available by the authors, without undue reservation.

## Supplementary material

Supplementary Table 1 Species data.

Supplementary Figure 1 Difference on top 20 dominant species importance.

Supplementary Figure 2 The result of correspondence analysis CA.

Supplementary Figure 3 ANOSIM test.

Supplementary Figure 4 Redundancy analysis RDA.

Supplementary Figure 5 Canonical correspondence analysis CCA.

## References

[B1] Backhaus L. Albert G. Cuchietti A. Jaimes Nino L. M. Fahs N. Lisner A.. (2021). Shift from trait convergence to divergence along old-field succession. J. Veg. Sci. 32, e12986. doi: 10.1111/jvs.12986

[B2] Bao S. (2000). Soil agrochemical analysis. 3rd ed. (China, Beijing: China Agriculture Press). doi: 10.1016/j.ecolmodel.2008.04.010

[B3] Bernard-Verdier M. Navas M. Vellend M. Violle C. Fayolle A. Garnier E. (2012). Community assembly along a soil depth gradient: contrasting patterns of plant trait convergence and divergence in a M editerranean rangeland. J. Ecol. 100, 1422–1433. doi: 10.1111/1365-2745.12003

[B4] Blanchard G. Ibanez T. Munoz F. Bruy D. Hely C. Munzinger J.. (2021). Drivers of tree community assembly during tropical forest post-fire succession in anthropogenic savannas. Perspect. Plant Ecol. Evol. Syst. 52, 125630. doi: 10.1016/j.ppees.2021.125630

[B5] Botta-Dukát Z. (2018). Cautionary note on calculating standardized effect size (SES) in randomization test. Community Ecol. 19, 77–83. doi: 10.1556/168.2018.19.1.8

[B6] Bu W. Zang R. Ding Y. (2014). Field observed relationships between biodiversity and ecosystem functioning during secondary succession in a tropical lowland rainforest. Acta Oecologica 55, 1–7. doi: 10.1016/j.actao.2013.10.002

[B7] Buzzard V. Hulshof C. M. Birt T. Violle C. Enquist B. J. (2016). Re-growing a tropical dry forest: functional plant trait composition and community assembly during succession. Funct. Ecol. 30, 1006–1013. doi: 10.1111/1365-2435.12579

[B8] Chave J. Coomes D. Jansen S. Lewis S. L. Swenson N. G. Zanne A. E. (2009). Towards a worldwide wood economics spectrum. Ecol. Lett. 12, 351–366. doi: 10.1111/j.1461-0248.2009.01285.x 19243406

[B9] Chen H. Nie Y. Wang K. (2013). Spatio-temporal heterogeneity of water and plant adaptation mechanisms in karst regions: a review. Acta Ecol. Sin. 33, 317–326. doi: 10.5846/stxb201112011836

[B10] Chen C. Wen Y. Ji T. Zhao H. Zang R. Lu X. (2022). Ecological Strategy Spectra for Communities of Different Successional Stages in the Tropical Lowland Rainforest of Hainan Island. Forests 13, 973. doi: 10.3390/f13070973

[B11] Chen K. Pan Y. Li Y. Cheng J. Lin H. Zhuo W.. (2023). Slope position- mediated soil environmental filtering drives plant community assembly processes in hilly shrublands of Guilin, China. Front. Plant Sci. 13. doi: 10.3389/fpls.2022.1074191 PMC9859686 36684746

[B12] Cheng X. Ping T. Li Z. Wang T. Han H. Epstein H. E. (2022). Effects of environmental factors on plant functional traits across different plant life forms in a temperate forest ecosystem. New For. 53, 125–142. doi: 10.1007/s11056-021-09847-0

[B13] Clarke K. R. (1993). Non-parametric multivariate analyses of changes in community structure. Aust. J. Ecol. 18, 117–143. doi: 10.1111/j.1442-9993.1993.tb00438.x

[B14] Czortek P. Orczewska A. Dyderski M. K. (2021). Niche differentiation, competition or habitat filtering? Mechanisms explaining co-occurrence of plant species on wet meadows of high conservation value. J. Veg. Sci. 32, e12983. doi: 10.1111/jvs.12983

[B15] Doležal J. Lanta V. Mudrák O. Lepš J. (2019). Seasonality promotes grassland diversity: Interactions with mowing, fertilization and removal of dominant species. J. Ecol. 107, 203–215. doi: 10.1111/1365-2745.13007

[B16] Frazer G. W. Canham C. D. Lertzman K. P. (2000). Gap light analyzer (GLA), Version 2.0: image-processing software to analyze true-color, hemispherical canopy photographs. Bulletin of the Ecological Society of America. 81, 191–197. doi: 10.2307/20168436

[B17] Freschet G. T. Dias A. T. C. Ackerly D. D. Aerts R. Van Bodegom P. M. Cornwell W. K.. (2011). Global to community scale differences in the prevalence of convergent over divergent leaf trait distributions in plant assemblages: Global patterns in plant species assembly. Glob. Ecol. Biogeogr. 20, 755–765. doi: 10.1111/j.1466-8238.2011.00651.x

[B18] Fu R. Dai L. Zhang Z. Hu G. (2023). Community assembly along a successional chronosequence in the northern tropical karst mountains, South China. Plant Soil 491, 317–331. doi: 10.1007/s11104-023-06118-z

[B19] Ge J. Berg. B. Xie Z. (2019). Climatic seasonality is linked to the occurrence of the mixed evergreen and deciduous broad-leaved forests in China. Ecosphere 10, e02862. doi: 10.1002/ecs2.2862

[B20] Guélat J. Jaquiéry J. Berset-Brändli L. Pellegrini E. Moresi R. Broquet T.. (2008). Mass effects mediate coexistence in competing shrews. Ecology 89, 2033–2042. doi: 10.1890/07-0905.1 18705388

[B21] Guo K. Fang J. Wang G. Tang Z. Xie Z. Shen Z.. (2020). A revised scheme of vegetation classification system of China. Chin. J. Plant Ecol. 44, 111. doi: 10.17521/cjpe.2019.0271

[B22] Han T. Ren H. Hui D. Zhu Y. Lu H. Guo Q.. (2023). Dominant ecological processes and plant functional strategies change during the succession of a subtropical forest. Ecol. Indic. 146, 109885. doi: 10.1016/j.ecolind.2023.109885

[B23] Huang L. (2012). Research on the “Great Leap Forward” Movement in Guangxi (Master's thesis) (Shaanxi Normal University).

[B24] Huang C. Xu Y. Zang R. (2021). Variation patterns of functional trait moments along geographical gradients and their environmental determinants in the subtropical evergreen broadleaved forests. Front. Plant Sci. 12. doi: 10.3389/fpls.2021.686965 PMC8311185 34322143

[B25] Huang Y. Zhang X. Zang R. Fu S. Ai X. Yao L.. (2018). Functional recovery of a subtropical evergreen-deciduous broadleaved mixed forest following clear cutting in central China. Sci. Rep. 8, 16458. doi: 10.1038/s41598-018-34896-5 30405174 PMC6220334

[B26] Hubbell S. P. (2001). The Unified Neutral Theory of Biodiversity and Biogeography (MPB-32) (Princeton University Press).

[B27] Jiang M. Yang X. Wang T. Xu Y. Dong K. He L.. (2021). A direct comparison of the effects and mechanisms between species richness and genotype richness in a dominant species on multiple ecosystem functions. Ecol. Evol. 11, 14125–14134. doi: 10.1002/ece3.8125 34707845 PMC8525171

[B28] Jiang Y. Zang R. Lu X. Huang Y. Ding Y. Liu W.. (2015). Effects of soil and microclimatic conditions on the community-level plant functional traits across different tropical forest types. Plant Soil 390, 351–367. doi: 10.1007/s11104-015-2411-y

[B29] Jung V. Violle C. Mondy C. Hoffmann L. Muller S. (2010). Intraspecific variability and trait-based community assembly. J. Ecol. 98, 1134–1140. doi: 10.1111/j.1365-2745.2010.01687.x

[B30] Kazakou E. Bumb I. Garnier E. (2022). Species dominance rather than complementarity drives community digestibility and litter decomposition in species-rich Mediterranean rangelands. Appl. Veg. Sci. 25, e12685. doi: 10.1111/avsc.12685

[B31] Kembel S. W. Cowan P. D. Helmus M. R. Cornwell W. K. Morlon H. Ackerly D. D.. (2010). Picante: R tools for integrating phylogenies and ecology. Bioinformatics 26, 1463–1464. doi: 10.1093/bioinformatics/btq166 20395285

[B32] Kohli B. A. Terry R. C. Rowe R. J. (2018). A trait-based framework for discerning drivers of species co-occurrence across heterogeneous landscapes. Ecography 41, 1921–1933. doi: 10.1111/ecog.03747

[B33] Kraft N. J. B. Valencia R. Ackerly D. D. (2008). Functional Traits and niche-based tree community assembly in an Amazonian forest. Science 322, 580–582. doi: 10.1126/science.1160662 18948539

[B34] Kröber W. Heklau H. Bruelheide H. (2015). Leaf morphology of 40 evergreen and deciduous broadleaved subtropical tree species and relationships to functional ecophysiological traits. Plant Biol. 17, 373–383. doi: 10.1111/plb.12250 25441614

[B35] Lepš J. De Bello F. (2023). Differences in trait-environment relationships: Implications for community weighted means tests. J. Ecol. 111, 2328–2341. doi: 10.1111/1365-2745.14172

[B36] Letcher S. Lasky R. Chazdon N. Norden S. Wright J. A. Meave E.. (2015). “Environmental Gradients and the Evolution of Successional Habitat Specialization: A Test Case with 14 Neotropical Forest Sites.” J. Ecol. 103, 1276–1290. doi: 10.1111/1365-2745.12435

[B37] Lhotsky B. Kovács B. Ónodi G. Csecserits A. Rédei T. Lengyel A.. (2016). Changes in assembly rules along a stress gradient from open dry grasslands to wetlands. J. Ecol. 104, 507–517. doi: 10.1111/1365-2745.12532

[B38] Li S. Cadotte M. W. Meiners S. J. Hua Z. Jiang L. Shu W. (2015). Species colonisation, not competitive exclusion, drives community overdispersion over long-term succession. Ecol. Lett. 18, 964–973. doi: 10.1111/ele.12476 26189648

[B39] Li Y. Zheng J. Wang G. Zhou J. Liu Y. Ha W. (2021). A study of functional traits of natural secondary forests and their influencing factors in different succession stages in karst areas: A case study of Dahei Mountain, Yunnan province. Acta Geosci. Sin., 42(3)397–406. doi: 10.3975/cagsb.2020.070902

[B40] Liang S. Lin H. Bao H. Yao Y. Jiang Y. Li Y.. (2020). Distribution pattern of trait-based community assembly for *Cyclobalanopsis glauca* in the Guilin karst mountainous areas, China. Trop. Conserv. Sci. 13, 194008292098027. doi: 10.1177/1940082920980279

[B41] Liu C. Huang Y. Wu F. Liu W. Ning Y. Huang Z.. (2021). Plant adaptability in karst regions. J. Plant Res. 134, 889–906. doi: 10.1007/s10265-021-01330-3 34258691

[B42] Long Q. Du H. Su L. Zeng F. Lian Z. Peng W.. (2023). Patterns in leaf traits of woody species and their environmental determinants in a humid karstic forest in southwest China. Front. Ecol. Evol. 11. doi: 10.3389/fevo.2023.1230819

[B43] López-Martínez J. O. Sanaphre-Villanueva L. Dupuy J. M. Hernández-Stefanoni J. L. Meave J. A. Gallardo-Cruz J. A. (2013). [amp]]beta;-diversity of functional groups of woody plants in a tropical dry forest in Yucatan. PloS One 8, e73660. doi: 10.1371/journal.pone.0073660 24040014 PMC3769343

[B44] Lusk C. H. Grierson E. R. P. Laughlin D. C. (2019). Large leaves in warm, moist environments confer an advantage in seedling light interception efficiency. New Phytol. 223, 1319–1327. doi: 10.1111/nph.15849 30985943

[B45] Lv Y. He H. Ren X. Zhang L. Qin K. Wu X.. (2022). High resistance of deciduous forests and high recovery rate of evergreen forests under moderate droughts in China. Ecol. Indic 144:109469. doi: 10.1016/j.ecolind.2022.109469

[B46] Ma Y. Huang Y. Zhao Y. Chi Y. Sharen T. Guo Y. (2021). Relationship between specific leaf area and water use efficiency of strain Populus simonii × P. euphratica in Ulanbuh Desert. J. Arid Land Resour. Environ. 35, 150–155. doi: 10.13448/j.cnki.jalre.2021.339

[B47] Mayfield M. Levine J. (2010). Opposing effects of competitive exclusion on the phylogenetic structure of communities. Ecology Letters 13, 1085–1093. doi: 10.1111/j.1461-0248.2010.01509.x 20576030

[B48] Marcilio-Silva V. Pillar V. D. Marques M. C. M. (2016). Functional turnover and community assemblage during tropical forest succession. Community Ecol. 17, 88–97. doi: 10.1556/168.2016.17.1.11

[B49] Marteinsdóttir B. Svavarsdóttir K. Thórhallsdóttir T. E. (2018). Multiple mechanisms of early plant community assembly with stochasticity driving the process. Ecology 99, 91–102. doi: 10.1002/ecy.2079 29121406

[B50] McIntyre S. Lavorel S. Landsberg J. Forbes T. D. A. (1999). Disturbance response in vegetation - towards a global perspective on functional traits. J. Veg. Sci. 10, 621–630. doi: 10.2307/3237077

[B51] Meilhac J. Deschamps L. Maire V. Flajoulot S. Litrico I. (2019). Both selection and plasticity drive niche differentiation in experimental grasslands. Nat. Plants 6, 28–33. doi: 10.1038/s41477-019-0569-7 31873193

[B52] Mudrák O. Janeček Š. Götzenberger L. Mason N. W. H. Horník J. De Castro I.. (2016). Fine-scale coexistence patterns along a productivity gradient in wet meadows: shifts from trait convergence to divergence. Ecography 39, 338–348. doi: 10.1111/ecog.01723

[B53] Muscarella R. Kolyaie S. Morton D. C. Zimmerman J. K. Uriarte M. (2020). Effects of topography on tropical forest structure depend on climate context. J. Ecol. 108, 145–159. doi: 10.1111/1365-2745.13261

[B54] Nock C. A. Vogt R. J. Beisner B. E. (2016). “Functional traits,” in eLS (John Wiley & Sons, Ltd). doi: 10.1002/9780470015902.a0026282

[B55] Nomura Y. Matsuo T. Ichie T. Kitayama K. Onoda Y. (2023). Quantifying functional trait assembly along a temperate successional gradient with consideration of intraspecific variations and functional groups. Plant Ecol. 224, 669–682. doi: 10.1007/s11258-023-01329-x

[B56] Pérez-Harguindeguy N. Díaz S. Garnier E. Lavorel S. Poorter H. Jaureguiberry P.. (20132013). New handbook forstandardised measurement of plant functional traits worldwide. Aust. J. Botany. 61, 167–234. doi: 10.1071/BT12225

[B57] Purschke O. Schmid B. C. Sykes M. T. Poschlod P. Michalski S. G. Durka W.. (2013). Contrasting changes in taxonomic, phylogenetic and functional diversity during a long-term succession: insights into assembly processes. J. Ecol. 101, 857–866. doi: 10.1111/1365-2745.12098

[B58] Qiao J. Zuo X. Yue P. Wang S. Hu Y. Guo X.. (2023). High nitrogen addition induces functional trait divergence of plant community in a temperate desert steppe. Plant Soil 487, 133–156. doi: 10.1007/s11104-023-05910-1

[B59] Scheffer M. Van Nes E. H. (2006). Self-organized similarity, the evolutionary emergence of groups of similar species. Proc. Natl. Acad. Sci. 103, 6230–6235. doi: 10.1073/pnas.0508024103 16585519 PMC1458860

[B60] Schmitt S. Trueba S. Coste S. Ducouret É. Tysklind N. Heuertz M.. (2022). Seasonal variation of leaf thickness: An overlooked component of functional trait variability. Plant Biol. 24, 458–463. doi: 10.1111/plb.13395 35120262

[B61] Schönbeck L. Lohbeck M. Bongers F. Ramos M. Sterck F. (2015). How do Light and Water Acquisition Strategies Affect Species Selection during Secondary Succession in Moist Tropical Forests? Forests 6, 2047–2065. doi: 10.3390/f6062047

[B62] Shivaprakash K. N. Ramesh B. R. Umashaanker R. Dayanandan S. (2018). Functional trait and community phylogenetic analyses reveal environmental filtering as the major determinant of assembly of tropical forest tree communities in the Western Ghats biodiversity hotspot in India. For. Ecosyst. 5, 25. doi: 10.1186/s40663-018-0144-0

[B63] Su Z. Li X. (2003). The types of natural vegetation in karst region of Guangxi and its classified system. Guihaia 23, 289–293. Available at: https://www.researchgate.net/publication/285751370.

[B64] Tilman D. (2004). Niche tradeoffs, neutrality, and community structure: A stochastic theory of resource competition, invasion, and community assembly. Proc. Natl. Acad. Sci. 101, 10854–10861. doi: 10.1073/pnas.0403458101 15243158 PMC503710

[B65] Uriarte M. Swenson N. G. Chazdon R. L. Comita L. S. John Kress W. Erickson D.. (2010). Trait similarity, shared ancestry and the structure of neighbourhood interactions in a subtropical wet forest: implications for community assembly. Ecol. Lett. 13, 1503–1514. doi: 10.1111/j.1461-0248.2010.01541.x 21054732

[B66] Vargas G. Brodribb T. J. Dupuy J. M. González-M R. Hulshof C. M. Medvigy D.. (2021). Beyond leaf habit: generalities in plant function across 97 tropical dry forest tree species. New Phytol. 232, 148–161. doi: 10.1111/nph.17584 34171131

[B67] Veress M. (2020). Karst types and their karstification. J. Earth Sci. 31, 621–634. doi: 10.1007/s12583-020-1306-x

[B68] Vialatte A. Bailey R. I. Vasseur C. Matocq A. Gossner M. M. Everhart D.. (2010). Phylogenetic isolation of host trees affects assembly of local Heteroptera communities. Proc. R. Soc B Biol. Sci. 277, 2227–2236. doi: 10.1098/rspb.2010.0365 PMC2880160 20335208

[B69] Violle C. Enquist B. J. McGill B. J. Jiang L. Albert C. H. Hulshof C.. (2012). The return of the variance: intraspecific variability in community ecology. Trends Ecol. Evol. 27, 244–252. doi: 10.1016/j.tree.2011.11.014 22244797

[B70] Violle C. Jiang L. (2009). Towards a trait-based quantification of species niche. J. Plant Ecol. 2, 87–93. doi: 10.1093/jpe/rtp007

[B71] Vleminckx J. Barrantes O. V. Fortunel C. Paine C. E. T. Bauman D. Engel J.. (2023). Niche breadth of Amazonian trees increases with niche optimum across broad edaphic gradients. Ecology 104, e4053. doi: 10.1002/ecy.4053 37079023

[B72] Wang L. He Y. Umer M. Guo Y. Tan Q. Kang L.. (2023). Strategic differentiation of subcommunities composed of evergreen and deciduous woody species associated with leaf functional traits in the subtropical mixed forest. Ecol. Indic. 150, 110281. doi: 10.1016/j.ecolind.2023.110281

[B73] Wang X. Sun S. Sedio B. E. Glomglieng S. Cao M. Cao K.. (2022a). Niche differentiation along multiple functional-trait dimensions contributes to high local diversity of Euphorbiaceae in a tropical tree assemblage. J. Ecol. 110, 2731–2744. doi: 10.1111/1365-2745.13984

[B74] Wang Y. Zhang L. Chen J. Feng L. Li F. Yu L. (2022b). Study on the relationship between functional characteristics and environmental factors in karst plant communities. Ecol. Evol. 12, e9335. doi: 10.1002/ece3.9335 36188516 PMC9486817

[B75] Wei Y. Wang Z. Liang W. Ma F. Han L. (2020). Response and adaptation of twig-leaf functional traits of Populus euphratica to groundwater gradients. Acta Bot. Boreali-Occident. Sin. 40, 1043–1051. doi: 10.7606/j.issn.1000-4025.2020.06.1043

[B76] Weiher E. Freund D. Bunton T. Stefanski A. Lee T. Bentivenga S. (2011). Advances, challenges and a developing synthesis of ecological community assembly theory. Philos. Trans. R. Soc B Biol. Sci. 366, 2403–2413. doi: 10.1098/rstb.2011.0056 PMC3130429 21768155

[B77] Wellstein C. Poschlod P. Gohlke A. Chelli S. Campetella G. Rosbakh S.. (2017). Effects of extreme drought on specific leaf area of grassland species: A meta-analysis of experimental studies in temperate and sub-Mediterranean systems. Glob. Change Biol. 23, 2473–2481. doi: 10.1111/gcb.13662 28208238

[B78] Wen Y. Zhou X. Wang L. Sun D. (2022). Vegetation survey, classification and the research and compilation of vegegraphy of Guangxi, China. J. Guangxi Acad. Sci. 38, 245–253. doi: 10.13657/j.cnki.gxkxyxb.20221019.004

[B79] Wu T. (2023). Study on leaf functional traits in different succession stages ofMaolan karst forest (Master's thesis) (Guizhou normal university).

[B80] Wu G. Chen D. Zhou Z. (2021). Contrasting hydraulic efficiency and photosynthesis strategy in differential successional stages of a subtropical forest in a karst region. Plants 10, 2604. doi: 10.3390/plants10122604 34961075 PMC8705339

[B81] Xu J. Chai Y. Wang M. Dang H. Guo Y. Chen Y.. (2018). Shifts in Plant Community Assembly Processes across Growth Forms along a Habitat Severity Gradient: A Test of the Plant Functional Trait Approach. Front. Plant Sci. 9. doi: 10.3389/fpls.2018.00180 PMC5818416 29497437

[B82] Yan B. Zhang J. Liu Y. Li Z. Huang X. Yang W.. (2012). Trait assembly of woody plants in communities across sub-alpine gradients: Identifying the role of limiting similarity. J. Veg. Sci. 23, 698–708. doi: 10.1111/j.1654-1103.2011.01384.x

[B83] Yguel B. Bailey R. Tosh N. D. Vialatte A. Vasseur C. Vitrac X.. (2011). Phytophagy on phylogenetically isolated trees: why hosts should escape their relatives. Ecol. Lett. 14, 1117–1124. doi: 10.1111/j.1461-0248.2011.01680.x 21923895

[B84] Zhang J. Swenson N. G. Liu J. Liu M. Qiao X. Jiang M. (2020). A phylogenetic and trait-based analysis of community assembly in a subtropical forest in central China. Ecol. Evol. 10, 8091–8104. doi: 10.1002/ece3.6465 32788963 PMC7417225

[B85] Zhang H. Yang Q. Zhou D. Xu W. Gao J. Wang Z. (2021). How evergreen and deciduous trees coexist during secondary forest succession: Insights into forest restoration mechanisms in Chinese subtropical forest. Glob. Ecol. Conserv. 25, e01418. doi: 10.1016/j.gecco.2020.e01418

[B86] Zhu Y. Wang X. Wang X. Deng M. (2016). Effect of slope aspect on the functional diversity of grass communities in the Loess Plateau. Acta Ecol. Sin. 36, 21. doi: 10.5846/stxb201505010900

