# Supplementary material for: Trait-based community assembly and functional strategies across three subtropical karst forests, Southwestern China
Source: Front Plant Sci. 2024 Sep 9;15:1451981. doi: 10.3389/fpls.2024.1451981 (PMC11417004; doi:10.3389/fpls.2024.1451981)
Supplement: Supplementary Table 1 — Species data. [file DataSheet1.docx]

Trait-based community assembly and functional strategies across three Subtropical Karst forests, Southwestern China

**Electronic Supplementary Materials**

**Appendix Species importance values (Supplementary Table 1;** **Supplementary Figure 1)**

Species importance value was used to qualify for the dominant position of species in forest system. It is calculated as the function of average of relative density, relative basal area, and relative frequency on a percentage basis from Liu et al. (2014). The formula was calculated as follows:

$$IV=\frac{(RD+RA+RF)}{3}\times100\%$$

where *IV* is the importance value, *RD* is the relative dominance, *RA* is the relative density, *RF* is the relative frequency.

**Supplementary Table 1.** The top 20 dominant species composition with importance value at each forest type

| **Deciduous forests** | | **Mixed forests** | | **Evergreen forests** | |
| --- | --- | --- | --- | --- | --- |
| **Species** | **IV (%)** | **Species** | **IV (%)** | **Species** | **IV (%)** |
| *Celtis sinensis* | 28.2 | *Cyclobalanopsis glauca* | 19.9 | *Cyclobalanopsis glauca* | 35.5 |
| *Mallotus repandus* | 10.1 | *Zelkova schneideriana* | 9.7 | *Mallotus philippensis* | 9.8 |
| *Choerospondias axillaris* | 9.1 | *Boniodendron minus* | 8.6 | *Pittosporum planilobum* | 6.1 |
| *Chimonanthus nitens* | 7.1 | *Mallotus philippensis* | 4.8 | *Decaspermum fruticosum* | 4.2 |
| *Cornus wilsoniana* | 4.8 | *Cinnamomum saxatile* | 4.1 | *Albizia julibrissin* | 3.8 |
| *Boniodendron minus* | 4.8 | *Pittosporum planilobum* | 3.9 | *Murraya exotica* | 2.7 |
| *Ficus microcarpa* | 4.3 | *Callicarpa bodinieri* | 3.4 | *Diplospora dubia* | 1.9 |
| *Clausena anisum-olens* | 3.9 | *Garcinia paucinervis* | 3.2 | *Flueggea suffruticosa* | 1.9 |
| *Sapium rotundifolium* | 2.8 | *Sapium rotundifolium* | 3.1 | *Ficus erecta* | 1.9 |
| *Mallotus philippensis* | 2.7 | *Radermachera sinica* | 2.7 | *Sinoadina racemosa* | 1.7 |
| *Sophora prazeri* | 2.3 | *Sinoadina racemosa* | 2.6 | *Cinnamomum saxatile* | 1.7 |
| *Murraya exotica* | 2.3 | *Celtis biondii* | 2.5 | *Cladrastis platycarpa* | 1.6 |
| *Sinoadina racemosa* | 1.8 | *Rhamnus lamprophylla* | 2.3 | *Loropetalum chinense* | 1.5 |
| *Vitex negundo* | 1.5 | *Bridelia fordii* | 2.2 | *Sapium rotundifolium* | 1.5 |
| *Celtis biondii* | 1.4 | *Lindera megaphylla* | 2.9 | *Celtis biondii* | 1.2 |
| *Pittosporum planilobum* | 1.4 | *Machilus calcicola* | 2.0 | *Decaspermum gracilentum* | 1.2 |
| *Radermachera sinica* | 1.2 | *Pistacia chinensis* | 1.8 | *Lithocarpus litseifolius* | 1.1 |
| *Photinia serrulata* | 1.2 | *Pteroceltis tatarinowii* | 1.5 | *Millettia pulchra* | 1.0 |
| *Canthium dicoccum* | 1.1 | *Ficus tinctoria* | 1.3 | *Alchornea trewioides* | 0.9 |
| *Ulmus pumila* | 1.0 | *Croton euryphyllus* | 1.2 | *Cudrania cochinchinensis* | 0.8 |

To better visualize difference on top 20 dominant species importance at each forest type, please see Supplementary Figure 1.

**
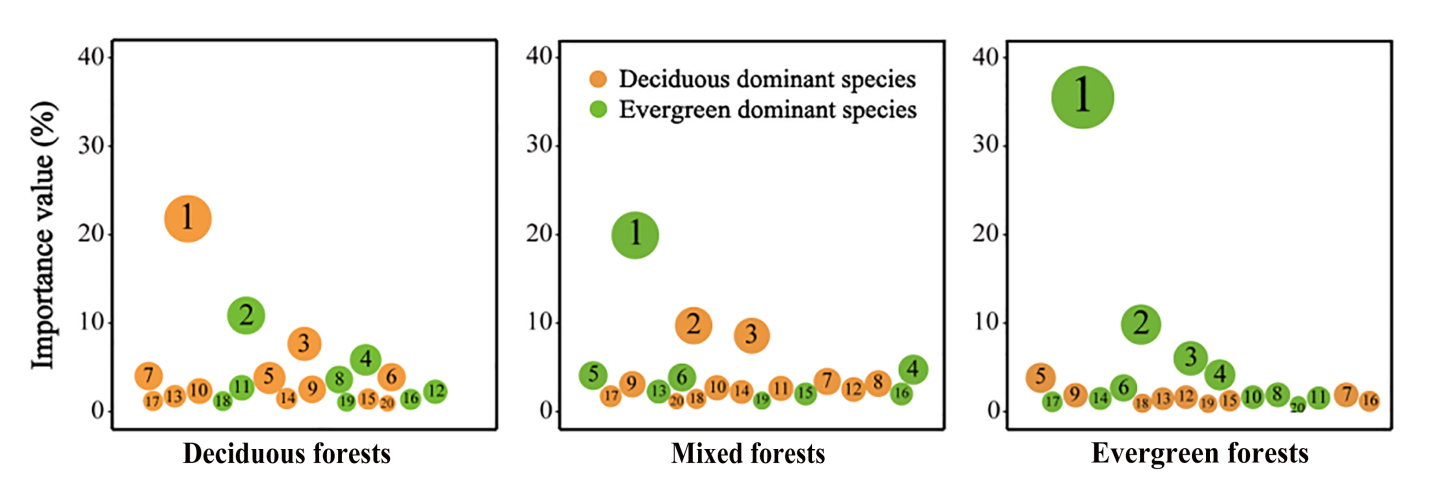
**

**Supplementary Figure 1.** Difference on top 20 dominant species importance at each forest type.

**Appendix** **Supplementary Figure 2**

Further, we performed Correspondence Analysis (CA) method using the species importance values data to distinguish this three forest types.

**
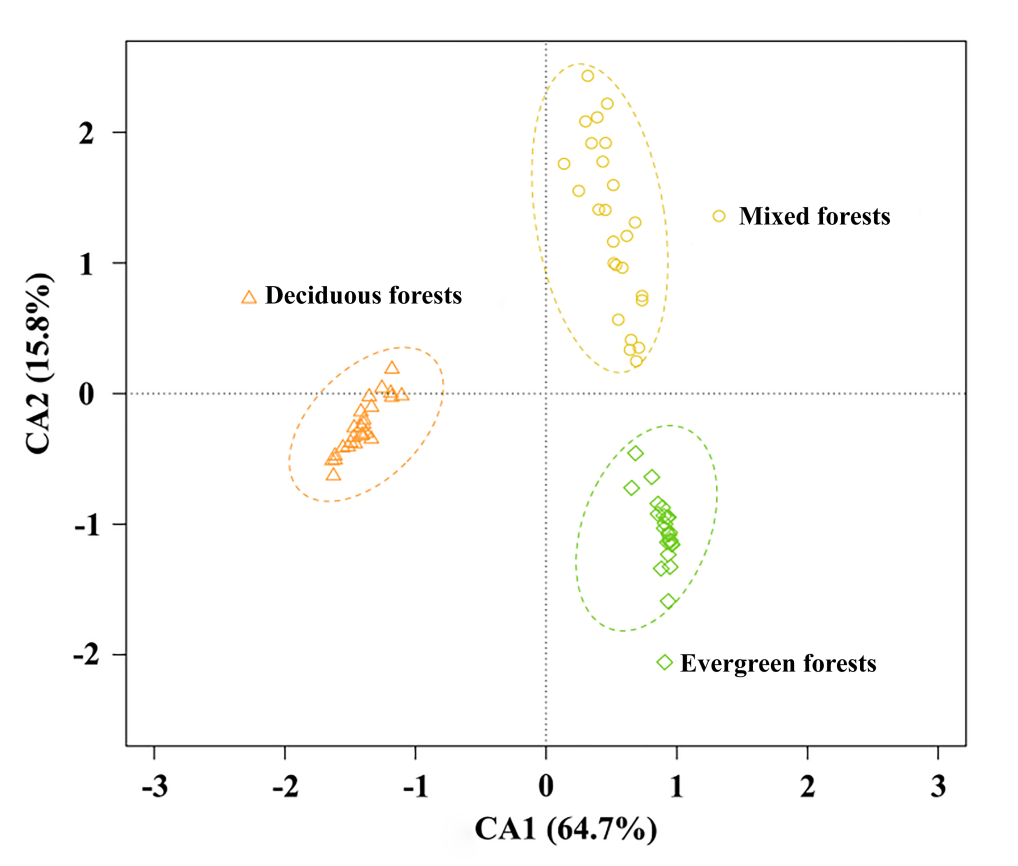
**

**Supplementary Figure 2.** Two-dimensional CA ordination diagram of plots.

Supplementary Figure 2 showed the sample plots and species can be divided into three distinct forest types.

**Appendix** **S****upplementary Figure 3**

Means of analysis of similarities (ANOSIM; Clarke, 1993) was performed by Bray-Curtis distances analysis to test whether there is a significant difference between two groups of sampling units. It is a robust non-parametric hypothesis for testing differences in resemblances among groups of samples (Somerfield et al., 2021). Each test in ANOSIM produces an R-statistic by calculating relating the mean difference in ranks between and within groups. R-statistic value distributes from −1 to +1, values close to 0 and negative reveal similarity between groups, while values close +1 reveal a strong dissimilarity between them (Clarke and Warwick 2001; Qureshi et al. 2019). All analyses were undertaken using the package ‘vegan’ version 2.0.6 (Oksanen et al., 2013) for the R software, version 4.0.2 (R Core Team, 2013).


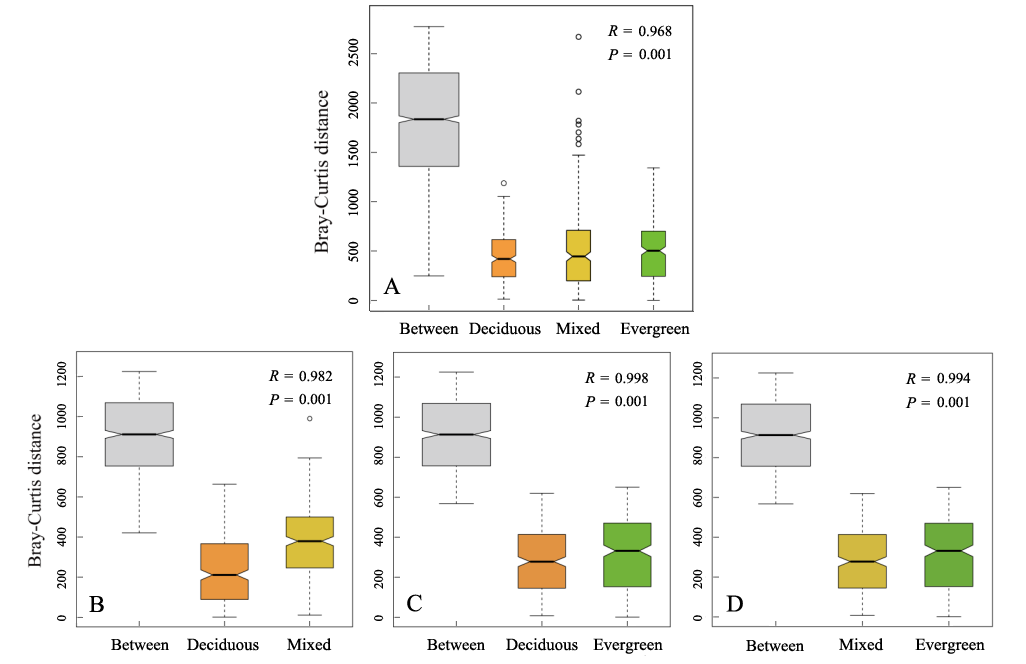


**Supplementary Figure 3.** Analysis of similarities between different forest types .

Supplementary Figure 3 clearly revealed significant difference in species composition between all forest types (*R*= 0.968, *p* = 0.001) (Supplementary Figure1A) and pair-wise forest types (*p* = 0.001, *R*=0.982, *R*=0.998 and *R*=0.994) between groups (Supplementary Figure 3B, C, D).

**Appendix** **Supplementary Figure 4**

We applied Redundancy analysis (RDA) based on environmental data matrix and traits matrix for sampling scale of 20m × 20m. The results showed five CWM_traits_ explained by environmental variables were 34.75% and 21.48% on the basis of the first two ordination axes (Supplementary Figure 4a and b). A total of 12 environmental factors, only soil Ca and soil thickness were observed no significant effect on five CWM_traits_ in the Supplementary Figure 4 (a) and (b) (permutation test 999 times using "envfit" function in R).


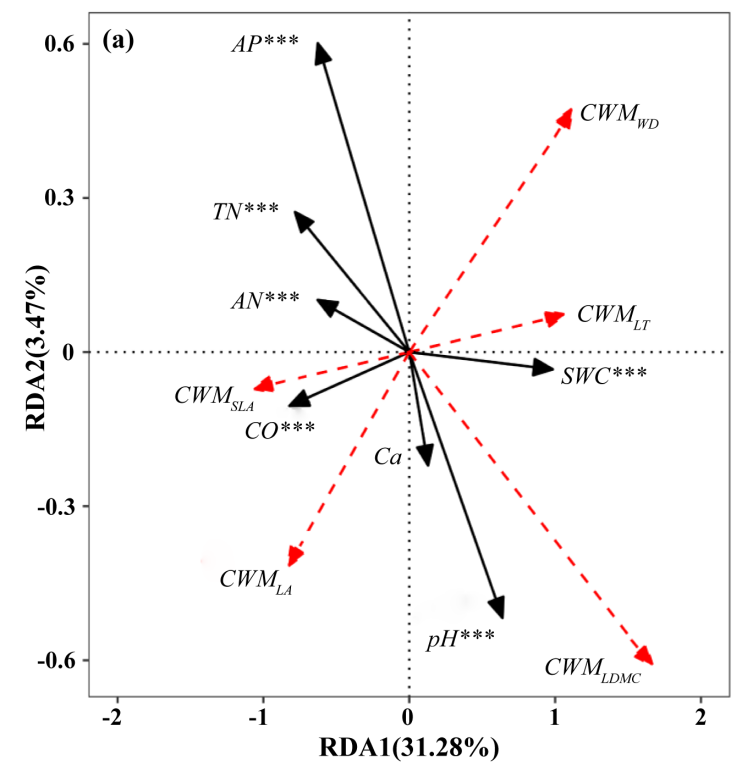

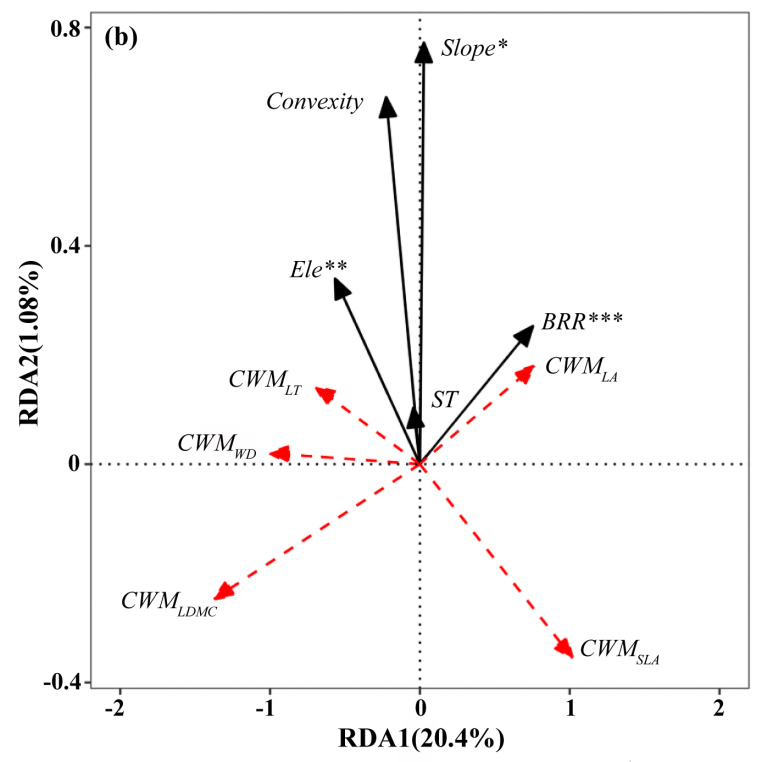


**Supplementary Figure 4.** The relationship between environmental variables and CWM_traits_ across three forest types by Redundancy analysis RDA ordination (a) and the relationship between terrain variables and CWM_traits_ across three forest types by Redundancy analysis RDA ordination. Notice: SWC, soil water content; ST, soil thickness; TN, total nitrogen; AN, available nitrogen; AP, available phosphorus; Ca, water-soluble calcium; CO, canopy openness; LA, leaf area; SLA, specific leaf area; LDMC, leaf dry matter content; LT, leaf thickness; WD, wood density; CWM, community-weighted mean; Ele, Elevation; BRR, rock-bareness ratio.

**Appendix Supplementary Figure 5**

We applied Canonical Correspondence analysis (CCA) with permutation test 999 times using "envfit" function in R based on environmental data matrix and traits matrix for sampling scale of 20m × 20m in three forest types. We found that SWC, AP, AN,CO and Slope significantly influenced five CWM_traits_ in deciduous forests while SWC, TN, AP, BRR and Slope had a strong impact on these five CWM_traits_ in evergreen forests, and CO, Slope and Elevation played an important role on mixed forests.


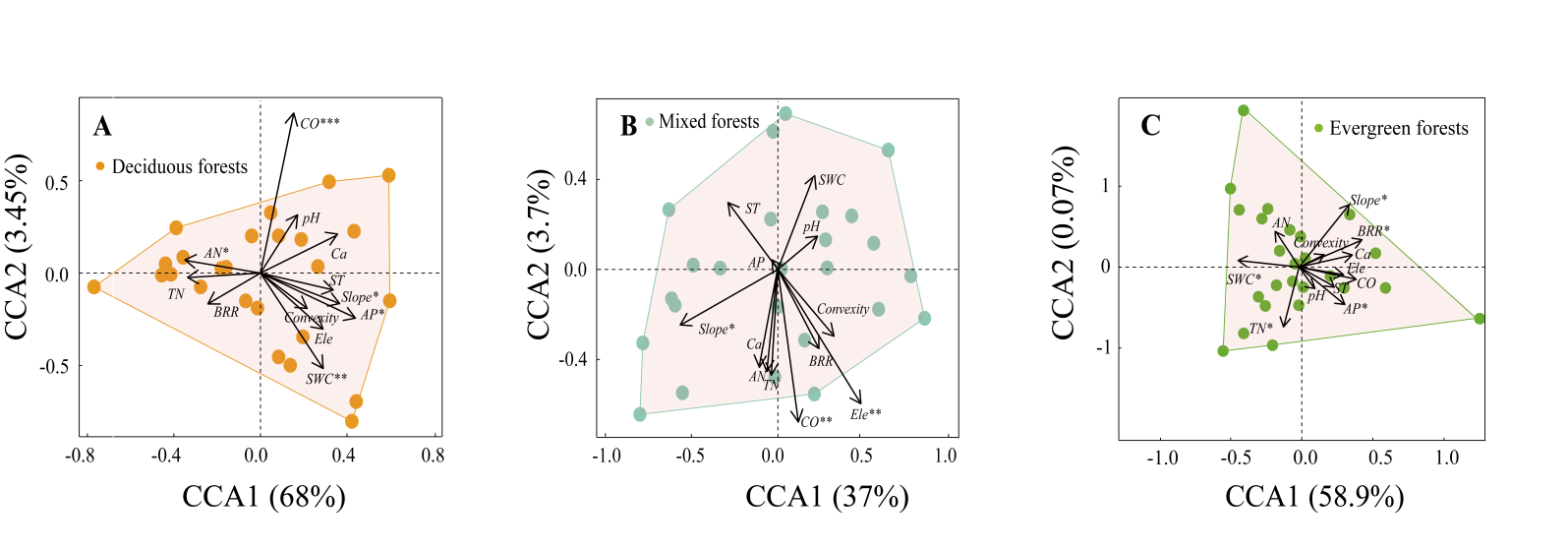


**Supplementary Figure 5.** The relationship between environmental variables and CWM_traits_ in three forest types by Canonical Correspondence analysis CCA ordination (**p*˂0.05; ***p*˂0.01; ****p*˂0.001). Notice: SWC, soil water content; ST, soil thickness; TN, total nitrogen; AN, available nitrogen; AP, available phosphorus; Ca, water-soluble calcium; CO, canopy openness; Ele, Elevation; BRR, rock-bareness ratio.

**References**

Clarke, K.R. (1993). Non-parametric multivariate analyses of changes in community structure. Australian Journal of Ecology 18, 117–143. https://doi.org/10.1111/j.1442-9993.1993.tb00438.x

Clarke, K.R., Warwick, R.M. (2001). A further biodiversity index applicable to species lists: variation in taxonomic distinctness. Marine ecology Progress series 216, 265–278.

Liu, C., Xiang, W., Lei, P., Deng, X., Tian, D., Fang, X., Peng, C. (2014). Standing fine root mass and production in four Chinese subtropical forests along a succession and species diversity gradient. Plant Soil 376, 445–459. https://doi.org/10.1007/s11104-013-1998-0

Oksanen, J., Blanchet, F.G., Kindt, R., Legendre, P., Minchin, P.R., O’hara, R.B., Simpson, G.L., Solymos, P., Stevens, M.H.H., Wagner, H. (2013). Community ecology package. R package version 2, 321–326.

Qureshi, H., Anwar, T., Arshad, M., Osunkoya, O.O., Adkins, S.W. (2019). Impacts of Xanthium strumarium L. invasion on vascular plant diversity in Pothwar Region (Pakistan). Annali di Botanica 9, 73–82.

Somerfield, P.J., Clarke, K.R., Gorley, R.N. (2021). A generalised analysis of similarities (ANOSIM) statistic for designs with ordered factors. Austral Ecology 46, 901–910. https://doi.org/10.1111/aec.13043
